# Cholestatic syndrome as initial manifestation of pancreatic metastasis of papillary thyroid carcinoma: case report and review

**DOI:** 10.20945/2359-3997000000215

**Published:** 2020-03-18

**Authors:** Mariana Yoshii Tramontin, Paulo Antônio Silvestre de Faria, Cristina Moreira do Nascimento, Cibele de Aquino Barbosa, Maria de Fátima Rei Pereira Barros, Aniela Rodrigues Gomes de Barros, Rafaela Cardoso de Carvalho, Antônio Kneipp Pitta de Castro, Fernanda Accioly de Andrade, Rossana Corbo, Fernanda Vaisman, Daniel Bulzico

**Affiliations:** 1 Unidade de Endocrinologia Oncológica Instituto Nacional de Câncer José Alencar Gomes da Silva Rio de Janeiro RJ Brasil Unidade de Endocrinologia Oncológica, Instituto Nacional de Câncer José Alencar Gomes da Silva (INCA), Rio de Janeiro, RJ, Brasil; 2 Serviço de Endocrinologia Hospital Federal da Lagoa Rio de Janeiro RJ Brasil Serviço de Endocrinologia, Hospital Federal da Lagoa, Rio de Janeiro, RJ, Brasil; 3 Divisão de Patologia Instituto Nacional de Câncer José Alencar Gomes da Silva Rio de Janeiro RJ Brasil Divisão de Patologia, Instituto Nacional de Câncer José Alencar Gomes da Silva (INCA), Rio de Janeiro, RJ, Brasil; 4 Seção de Cirurgia Abdominopélvica Instituto Nacional de Câncer José Alencar Gomes da Silva Rio de Janeiro RJ Brasil Seção de Cirurgia Abdominopélvica, Instituto Nacional de Câncer José Alencar Gomes da Silva (INCA), Rio de Janeiro, RJ, Brasil; 5 Serviço de Cirurgia Geral e Oncológica Hospital Central da Polícia Militar Rio de Janeiro RJ Brasil Serviço de Cirurgia Geral e Oncológica, Hospital Central da Polícia Militar, Rio de Janeiro, RJ, Brasil; 6 Faculdade de Medicina Universidade Federal do Rio de Janeiro Rio de Janeiro RJ Brasil Pós-graduação em Endocrinologia da Faculdade de Medicina da Universidade Federal do Rio de Janeiro (UFRJ), Rio de Janeiro, RJ, Brasil

## Abstract

Most papillary thyroid carcinomas (PTC) harbor excellent prognosis. Although rare, distant metastases normally occur in lungs and/or bones. Here we describe a rare case of pancreatic metastasis presenting with rapid onset cholestatic syndrome. A literature review was also performed. A 73-year-old man with a high risk PTC was submitted to total thyroidectomy (TT) followed by radioiodine therapy. After initial therapy, he persisted with progressive rising serum thyroglobulin levels but with no evidence of structural disease. Recently, the patient presented with a rapid onset and progressive cholestatic syndrome. A 4 cm lesion in pancreas was identified, with echoendoscopy fine-needle aspiration biopsy (FNAB) confirming a pancreatic metastasis from PTC. The patient was submitted to a successful pancreaticoduodenectomy. Pancreatic metastases of PTC are rare and few long-term follow-up data are available to guide management. Fourteen cases were former reported, mean age was 65.7 years-old with mean time between PTC and pancreatic metastasis diagnosis of 7.9 years. Nine of them had another distant metastasis, nine were diagnosed by FNAB and just two received sorafenib.

## INTRODUCTION

Thyroid cancer is the most common endocrine malignancy. Incidence has almost tripled since 1970, representing 3.1% of all new cancer cases but being responsible for just 0.3% of all cancer deaths in the United States according to SEER (Surveillance, Epidemiology, and End Results Program) database. Around 90% is derived from follicular cells and PTC is the most prevalent histological type (1). PTC generally has good prognosis expressed by 98% of overall survival rate. However up to 2.3 – 4.3% presents with distant metastasis at diagnosis (2,3).

Distant metastasis is the most important death-risk prognostic factor, with stage IV patients presenting with disease-specific survival at 5 and 10 years of 65% and 45% respectively (3). Lungs, bones, thoracic lymph nodes and nervous system are the most common organs involved (4). Unusual metastatic sites have been described in skin (5), pancreas (6), eye (7), kidney (8), adrenal (9), breast (10) and liver (11), but they do not always represent an additional negative prognostic factor for disease outcome (12).

Metastatic pancreatic tumors are rare and account for less than 4% of all pancreatic malignancies (13). The most common primary neoplasm is the kidney, with PTC metastases being extremely rare. Fourteen cases have been described so far, occurring up to 15 years after initial therapy. The treatments modalities described varied from surgery, chemotherapy, tyrosine kinase inhibitors (TKI), and active surveillance (6,10,14-24). Regarding survival, the benefit of metastasectomy has been observed for renal cell carcinoma metastases (25). About PTC, of the 7 cases submitted to metastasectomy and who had follow-up information, 4 died of the disease, 1 died of acute myocardial infarction, 1 had disease progression and 1 was alive at 36 months. Here we report one case of pancreatic metastasis from PTC initially presented as cholestatic syndrome with successful duodenopancreatectomy.

## CASE DESCRIPTION

A 73-year-old man, presented in 2012 with a large goiter, and suspicious lymph-nodes palpable in lateral neck. He was submitted to total thyroidectomy (TT) with central and right lateral lymphadenectomy (levels I-V) in 2012. Pathology study revealed a 5.5 cm classical papillary carcinoma with extra-thyroidal extension to cervical muscles, neoplastic emboli to lymphatics vessels, and gross invasion of the internal jugular vein. Metastases to 24 of 25 right cervical lymph nodes were observed – pT4aN1bMx (AJCC/TNM 8^th^ edition). Initial non-stimulated thyroglobulin (Tg) was of 5.81 ng/mL, with undetectable anti-Tg. The initial postoperative images (cervical ultrasound and chest computed tomography) showed no suspicious findings. After surgery the patient received radioiodine therapy (RAI) with 200 mCi of ^131^I. Post-therapy whole-body scan (WBS) was positive in right hemithorax although no structural disease was evident in thorax computed tomography (CT). During the first five years of follow-up he persisted with rising serum Tg levels but with no correspondent structural image in neck ultrasound, chest CT, or radioiodine WBS. Finally, in 2016 ^18^F-FDG PET revealed suspicious right posterior neck (SUV 7.7) and right supraclavicular lymph-nodes (SUV 15.9). An additional surgical neck dissection removed metastatic lymph nodes in August 2016.

Despite the additional surgical treatment, during the past year he evolved again with rapid rising of Tg levels (Tg: 32.8 ng/mL, TSH: 0.1 mU/L) and again with unidentified structural loco-regional disease and ^18^F-FDG PET was requested.

In January 2018, while waiting for ^18^F-FDG PET, the patient actively sought out the outpatient clinic because of altered liver biochemistry: aspartate aminotransferase: 210 UI/L (normal range: < 40 UI/L), alanine aminotransferase: 858 UI/L (reference range < 41UI/L), alkaline phosphatase: 173 UI/L (reference range: 40-129 UI/L), gamma glutamyl transferase: 739 UI/L (reference range < 60 UI/L). Bilirubin was normal (total bilirubin: 0.9 mg/dL, direct bilirubin: 0.5 mg/dL) and the patient was asymptomatic. Drug-induced hepatoxicity and viral hepatitis were excluded. One week later, hepatogram was repeated (AST: 240, ALT: 713, GGT: 1298, total bilirubin: 1.5, direct bilirubin: 0.9), and the patient began to present jaundice, nausea, anorexia, and fatigue. Weight loss started to become evident, jaundice worsened and next week laboratory showed AST: 261, ALT: 687, GGT: 1150, total bilirubin: 10.2, direct bilirubin: 8.2, albumin and prothrombin time were normal.

The ^18^F-FDG PET revealed a 2.8 cm expansive lesion in the head of the pancreas (SUV 10.6) in contact with duodenum, presenting partially defined limits with marked dilatation of biliary tract. At that occasion, Tg was of 281.3 ng/mL. He was referred to a CT-guided external percutaneous biliary drainage and fine-needle endoscopic ultrasound biopsy. Cytology study was consistent with metastatic PTC. Cell-block immunohistochemistry was positive to TTF-1.

After multidisciplinary discussion, in April 2018 the patient underwent a pancreaticoduodenectomy performed by an extended subcostal incision at Hospital Central da Polícia Militar. The pancreatogastrostomy was the technique of choice for pancreatic reconstruction after finding a soft pancreatic stump and non-dilated main duct. Double omental flap around the anastomosis was performed. The hepaticojejunostomy and gastroentero anastomosis was made in single loop and a cavity drain was setted up in the right flank. The transparietal drain was removed in the 10^th^ day after surgery. Patient had a favorable evolution getting hospital discharge in the 13^th^ day post operative without any complications. The pathology study described an infiltrative papillary architectural pattern carcinoma of 3.5 x 3.0 cm, compromising pancreatic tissue, presenting morphological characteristics compatible with metastatic PTC. Immunohistochemistry study was positive for Tg. After surgery, suppressed thyroglobulin was 241.3 ng/mL. Sorafenib was initiated in October, 2018 at full dosage (800 mg q.d.) and after one month the dose was reduced to 600 mg q.d. due to palmar-plantar erythrodysesthesia syndrome. Imaging studies show stable disease during sorafenb treatment. The patient is currently asymptomatic and attending outpatient clinics under surveillance at Instituto Nacional de Câncer José Alencar Gomes da Silva e Hospital Federal da Lagoa.

## DISCUSSION

Here we describe a case of pancreatic metastasis from PTC. Occurrence is rare, moreover is the first time that altered liver biochemistry and typical symptoms of cholestatic syndrome are the initial presentation. Of the reported cases, 35% describe isolated abdominal pain as initial symptom and 28% asymptomatic elevated Tg (6,14). Even thought the clinicopathologic features of unusual metastasis in PTC are not well established (12), previous known characteristics of poor prognosis and high risk of general distant metastasis in our case included older age and male gender additionally to high risk of recurrence, TNM classification (7th TNM: IVc, 8th TNM: IVb) and the development of radioactive iodine refractoriness.

Many of PTC metastasizes to regional lymph nodes but when the metastases are distant, lung and bone are the compromised sites. First described by Sugimura and cols. (15), metastases of PTC to pancreas are extremely rare, as evidenced by just 14 previously published cases (6,10,14-23). We performed a PubMed based research for previous reported cases using the following terms: “pancreatic metastasis AND thyroid cancer” during 1991 and July 2019. Only articles originally published in English language were included. Fourteen cases were found and are summarized in Table 1. Incidence of pancreatic metastasis was 1.8 times higher in male gender and from the 12 cases that reported histology, 7 were PTC, 3 tall cell variant and 2 follicular cell variant. Mean diagnosis age was 65.7 (39-84), 7.9 years after TT. About mortality risk, 5 were T4, 10 had lymph node involvement, 6 had other distant metastasis at the time of pancreatic metastasis but another 3 cases discovered it after. They comprised lung (8 cases), bone (4), liver (2), brain (1), adrenal (1) and omentum (1).

**Table 1:** Previous cases of pancreas metastasis from papillary thyoid carcinoma

Author	Ref	Year	Gender	Age	Time	Histology	Initial therapy	RAI	Stage	N	M	Presentation	Diagnosis	FDG-PET	Surgery	BRAFV600e	Follow-up
Sugimura	15	1991	F	39	7	PTC	Lob	No	N/A	No	N/A	Itching + jaundice	Surgery	N/A	Yes*	N/A	N/A
Jobran	16	2000	M	53	1.25	TCPTC	TT+ ND + laryngectomy +esophagectomy.	Yes	T4N1M0	Yes	L + B	Abdominal pain	CT-FNA	N/A	PD	N/A	Died 2 weeks after chemotherapy
Meyer	17	2006	M	67	5	PTC	TT + ND. Cervicotomy. EBRT. Retinoids.	Yes	T4N0M0	No	A+ L+Lv	Anaemia + bleeding	EUS-FNA	N/A	Partial PD	N/A	Died due to progressive disease 4.5 years after DP
Siddiqui	18	2006	M	69	7	TCPTC	TT + ND. EBRT.	Yes	T4N1bM0	Yes	L	Abdominal pain	EUS-FNA	positive	PD	N/A	Progression of lung disease at 24 months post-PD
Angeles-angeles	10	2009	M	72	N/A	PTC	No	N/A	N/A	N/A	B + Br	Abdominal pain	Surgery	N/A	DP	N/A	N/A
Borschitz (1)	19	2010	F	44	10	FVPTC	TT . Cervicotomy. EBRT	Yes	T3N1aM0	Yes	No	Tg. Asymptomatic	Surgery	positive	Enucleation	No	N/A
Borschitz (2)	19	2010	M	61	15	PTC	TT. EBRT. Cervicotomy. Metastasectomy.	Yes	T3N1M0	Yes	L + B	Tg. Asymptomatic	Surgery	positive	DP	Yes	Died due to progressive disease 3 years after DP
Chen	20	2010	M	82	N/A	PTC	TT.	Yes	N/A	Yes	N/A	Recurrent pancreatitis	EUS-FNA	N/A	N/A	N/A	N/A
Alzahrani	21	2012	M	55	7	PTC	TT+ ND. Cervicotomy. EBRT.	Yes	T4aN1bM0	Yes	L+Lv+ B+O	Tg. Asymptomatic	EUS-FNA	positive	No	Yes	Died due to progressive disease 20 months after pancreatic metastasis diagnosis
Tunio	22	2013	F	67	7	FVPTC	TT + ND.	Yes	T2N1cM0	Yes	L	Abdominal pain	CT-FNA	N/A	PD	Yes	Alive at 36months after PD
Xiao-Ou Li	23	2014	M	66	11	PTC	Lob. Cervicotomy.	N/A	N/A	Yes	No	Physical exam	Surgery	positive	DP	N/A	Died of acute myocardial infarction 5 months after DP
Davidson	6	2017	F	84	2	TCPTC	TT + ND.	Yes	T3N1bM0	Yes	No	Tg. Asymptomatic	EUS-FNA	positive	No	Yes	Surveillance
Murakami	14	2018	F	80	13	PTC	TT + ND.	N/A	T4aN1bM0	Yes	L	Tg. Asymptomatic	EUS-FNA	positive	DP	Yes	Died due to progressive disease 11 months after DP
CHO	24	2018	M	81	10	PTC	TT Cervicotomy	Yes	N/A	Yes	L	PET-CT. Asymptomatic.	EUS-FNA	Positive	No	N/A	Systemic treatment will be attempted

* Type of surgery not specified in the original article. Age: age at pancreatic diagnosis; Time: time between thyroidectomy and pancreatic metastasis diagnosis (years); Stage: according to UICC; RAI: radioiodine therapy, N: lymph-node disease at pancreatic metastasis diagnosis; M: distant metastases; N/A: not available; M: male; F: female; PTC: papillary thyroid carcinoma; TCPTC: tall cell PTC variant; FVPTC: follicular variant of PTC; TT: total thyroidectomy; Lob: lobectomy; ST: subtotal thyroidectomy; ND: neck dissection; EBRT: external beam radiation therapy; L: lung; B: Bone; A: adrenal; Lv: liver; Br: brain; O: omentum; Tg: high levels of thyroglobulin; CT-FNA: computer tomography guided fine-needle aspiration; EUS-FNA: Endoscopic ultrassound-guided FNA; PD: pancreaticoduodenectomy; DP: distal pancreatectomy.

Metastasis represent 1,8% to 4% of pancreatic masses and are generally related to kidney, skin, lung and breast malignancies (26,27). Differently from the usual pattern, 98% of pancreatic metastasis manifest as single lesions (27), being a potential diagnostic pitfall. Although not determinative, metastatic lesions are less likely with increasing age, presence of symptoms (abdominal pain, diarrhea, or weight loss), elevated bilirubin and arterial invasion on imaging when compared with pancreatic adenocarcinoma (28). Even though, our patient was an aged man with expressive weight loss and conjugated hyperbilirubinemia.

Since non-invasive methods are not accurate enough, endoscopic ultrasound-guided fine-needle biopsy has become the preferred method for diagnosis of pancreatic masses. Reported sensitivity and specificity for metastatic lesions are 84.9% and 100% respectively (28). Cytology generally displays cytomorphologic features similar to those of the primary neoplasm (27).

Most previously reported patients have known advanced cancer with other organs metastases at the time pancreatic lesion is discovered (10,14,16-19,21,22). Even though time for diagnosis was variable from one month to fifteen years after TT, and as an exception, in one case PTC diagnosis was done after pancreatectomy (10). The majority of patients with PTC and pancreatic metastases could be defined as iodine refractory, since biological and clinical features such as the progressive disease pattern despite previous high dose ^131^I therapy, and high ^18^F-FDG uptake were commonly found. Our case demonstrated positivity of the pancreatic lesion at ^18^F-FDG, as the same way as 100% of reported cases that performed it. Despite this, no anaplastic features were found in our pathology report.

For most metastatic well differentiated thyroid cancers, TT and radioiodine followed by TSH suppression are the gold standard therapy. However, in up to 30% of these cases iodine refractoriness will occur during follow-up (29,30). In this setting, 10-year survival reduces to 10% (30), and therapy approaches must be individualized according to factors such as disease volume, burden of disease, and symptomatic progression (31). Localized therapy directed to single metastasis patients must be the first line approach whenever possible in these patients. In a retrospective study, HO PAK and cols. (32) analyzed 47 metastasectomies (none in the pancreas) and found cumulative survival of 50.2 ± 12.5% at 10 years after the first metastasectomy. Experienced surgeons in specialized centers are able to perform pancreaticoduodenectomy with mortality rates as low as 2% in a population composed of more than 70% of aged over 75-years-old (33). Although surgical resections of pancreatic metastasis are not well established, consideration has to be done in cases of isolated metastasis or very symptomatic patients, once metastasectomy could reduce tumor burden and improve both survival and quality of life. Ten of the fourteen cases reported managed the pancreatic metastasis with surgery (Table 1). For patients with multiple metastases, especially those with symptomatic progression, TKI are the current treatment of choice with well described improvement in disease-progression free interval (34,35). To date, sorafenib and lenvatinib are the only two FDA approved molecules for this purpose. In the pancreatic metastasis, 2 cases reported the use of sorafenib, one evolved with disease progression (21) and the other had good response (22).

In conclusion, this is the first time a pancreatic metastasis of PTC presents as cholestatic syndrome. Despite rare, secondary malignancies in the pancreas have to be considered and endoscopic ultrasound is an important diagnostic tool that can lead to correct diagnosis and proper management. More experience is needed in the treatment of these metastases, meanwhile, TKI and duodenopancreatectomy should be considered.
